# Inactivation of Zona Incerta Blocks Social Conditioned Place Aversion and Modulates Post-traumatic Stress Disorder-Like Behaviors in Mice

**DOI:** 10.3389/fnbeh.2021.743484

**Published:** 2021-10-21

**Authors:** Hong Zhou, Wei Xiang, Mengbing Huang

**Affiliations:** Department of Physiology, School of Basic Medicine, Tongji Medical College, Huazhong University of Science and Technology, Wuhan, China

**Keywords:** zona incerta, tetanus toxin light chain, social conditioned place aversion, repeated social defeat stress, post-traumatic stress disorder

## Abstract

Zona incerta (ZI), a largely inhibitory subthalamic region connected with many brain areas, has been suggested to serve as an integrative node for modulation of behaviors and physiological states, such as fear memory conditioning and aversion responses. It is, however, unclear whether ZI regulated the repeated social defeat stress (RSDS)-induced social conditioned place aversion (CPA) and post-traumatic stress disorder (PTSD)-like behaviors. In this study, the function of ZI was silenced via bilateral injection of tetanus toxin light chain (Tet-tox), a neurotoxin that completely blocks the evoked synaptic transmissions, expressing adeno-associated viruses (AAVs). We found ZI silencing: (1) significantly blocked the expression of RSDS-induced social CPA with no effect on the innate preference; (2) significantly enhanced the anxiety level in mice experienced RSDS with no effect on the locomotion activity; (3) altered the PTSD-associated behaviors, including the promotion of spatial cognitive impairment and the preventions of PPI deficit and social avoidance behavior. These effects were not observed on non-stressed mice. In summary, our results suggest the important role of ZI in modulating RSDS-induced social CPA and PTSD-like behaviors.

## Introduction

The zona incerta (ZI) is a subthalamic region with extensive connections throughout the brain. It plays diverse roles in processing sensory information, regulating behaviors, conveying motivational states, and participating in neural plasticity (Zhang and van den Pol, 2017; Zhou et al., 2018; Zhao et al., 2019; Hormigo et al., 2020). For instance, activation of the midcingulate cortex Cg2 to ZI circuit alleviates neuropathic pain (Hu et al., 2019). ZI bidirectionally modulates defensive behaviors via different of its neuron subpopulations (Wang et al., 2019). Activation of the GABAergic neurons in the rostral part of ZI suppresses both noisy sound-induced flight and conditioned freezing response via their projections to the periaqueductal gray (Chou et al., 2018), while inactivation of the parvalbumin-positive neurons in the ventral part of the medial ZI or silencing all ZI neurons largely impairs fear conditioning memory acquisition and remote retrieval (Zhou et al., 2018). ZI was, therefore, important in modulating responses to aversive stimuli.

Social stressors are known to control affective-like behavioral responses across a wide variety of mammalian species (Fuchs and Flügge, 2002; Toth and Neumann, 2013; Menard et al., 2017). A large number of studies have been employed to discover the neurobiological impact of social defeat exposures as well as the bio-psycho-social factors moderating the response to social defeat in the past decades, during which several animal models of social defeat stress have been developed, including the sensory contact model (Kudryavtseva et al., 1991; Mahfoz, 2019), the chronic psychosocial stress model (Bartolomucci et al., 2001; Haffner-Luntzer et al., 2019), the social disruption model (Stark et al., 2001), and the repeated social defeat stress (RSDS) model (Berton et al., 2006). In the standardized RSDS model, C57BL/6J mice (intruder) are repeatedly subjected to bouts of social defeat by a larger CD-1 mouse (resident aggressor). Although each individual defeat lasts only 5–10 min, the defeated mouse is subjected to maintain psychological stress from sensory (visual, auditory, and olfactory) interaction with the aggressor for the duration of the experiment through a clear perforated divider in a shared home cage (Golden et al., 2011; Hammels et al., 2015).

Repeated exposures to social defeat stress has been reported to cause a variety of molecular, physiological, and behavioral changes (Bondar et al., 2018; Miyanishi et al., 2021; Quessy et al., 2021). In particular, animals suffered with RSDS exhibit loss of self-esteem, robust and sustained social avoidance, cognitive deficit, anxiety-, and depression-like phenotypes (Björkqvist, 2001; Wagner et al., 2013; Kosuge et al., 2021). These impaired emotional behaviors associated with traumatic social memory reflected a post-traumatic stress disorder (PTSD)-like state, which develops after exposure to a serious threatening event including RSDS results in significant, persistent negative changes in mood, arousal, and cognition (Browne et al., 2018). In addition to the PTSD-like behaviors, it is not known whether RSDS as a social aversive stimulus, can induce the formation of conditioned place preference or aversion (CPP or CPA) by combining the paradigm of Pavlovian learning. It is also unclear whether ZI involved in the modulation of RSDS-associated behaviors, such as CPA, and PTSD-like behaviors.

In the present study, we hypothesized that ZI was involved in regulating the RSDS-induced social CPA and PTSD-like behaviors. Using RSDS, we established a mouse social CPA model; the function of ZI was silenced by microinjection of adeno-associated viruses (AAVs) expressing tetanus toxin light chain (Tet-tox) and/or green fluorescent protein (GFP); and behaviors include the locomotion activity, anxiety level, spatial cognition, social interaction, and prepulse inhibition (PPI) were examined with corresponding behavioral paradigms.

## Materials and Methods

### Mice

Male C57BL/6J mice (5 weeks) and male CD1 (ICR) mice (4 months) were purchased from SPF Biotechnology Co., Ltd., (Beijing, China). The C57 mice were housed in groups of five, while the CD1 mice were housed in single. All mice were maintained on a 12 h light/dark cycle (8:30–20:30) with constant temperature (21 ± 2°C) and *ad libitum* access to food and water except during behavioral tests, and were allowed 1 week of acclimation to the housing facilities before the start of experiments. Mice were divided into four groups: non-stressed GPF group (with AAV-hSyn-GFP (AAV-GFP) injection and without RSDS, *n* = 9), non-stressed Tet-tox group [with AAV-CAG-Tet-tox-2A-GFP (AAV-Tet-tox) injection and without RSDS, *n* = 8], stressed GFP group (with AAV-GFP injection and RSDS, *n* = 10), stressed Tet-tox group (with AAV-Tet-tox injection and RSDS, *n* = 10). This study was approved by the Institutional Animal Care and Use Committee of the Huazhong University of Science and Technology.

### Aggression Screening

Aggression screening was performed as previously described for the resident-intruder (RI) test (Flanigan et al., 2020). After a minimum of 1 week of habituation to home cages, CD1 mice were exposed daily to a novel C57BL/6 J intruder for 10–15 min over three consecutive days. Presentation of each intruder was performed daily in the cages of experimental mice from 12:00 to 15:00 under white light condition. Cage covers were removed for unhindered viewing and videotaping during the RI sessions. All RI sessions were video recorded with a digital color video camera. Three aggression indicators were recorded: (1) the initial attack latency; (2) the total attack duration; (3) the number of attacks. Resident mice that initiated aggression during all three screening sessions were used for the stressor in subsequent experiments, whereas the mice that showed no aggression during any screening session were eliminated. Aggression screening was halted if an intruder showed any signs of injury in accordance with the published protocols (Golden et al., 2016).

### Virus Injection

Mice were anesthetized with chloral hydrate (400 mg/kg, i.p.) before surgery. All of the surgical tools and materials were autoclaved. The AAV-Tet-tox (Shanghai Qiji Biotechnology Co., Ltd., China) or AAV-GFP (Shanghai Genechem Co., Ltd., China) was delivered bilaterally to the ZI of mice (AP, −1.5 mm from the bregma; ML, ± 1.5 mm from the midline; DV, −4.2 mm from the dura). Four hundred nanoliters (nl) of virus was injected into each side at a rate of 2 nl/s. After injection, the needle was left in the brain for an additional 5 min before being slowly withdrawn in order to prevent the virus from leaking out. Mice were housed for 2 weeks before the start of social defeat to allow for recovery from surgery and sufficient viral expression. The virus injection sites were identified using Fluorescent inverted microscope (IX71, OLYMPUS, Japan).

### Social Conditioned Place Aversion

The model of social CPA was established according to the published protocols with slight modification (Golden et al., 2016). This task consisted of three phases: pre-test, acquisition (conditioning) and test. Briefly, mice were acclimated to the testing facility for 1 h before all tests. All phases were conducted under red light and sound-attenuated conditions. The CPA apparatus consisted of two unique conditioning chambers (20 × 30 × 30 cm^3^) with differences in visual (black or white wall) and tactile (smooth or rough floor) cues and divided by a sliding door. During the pre-test phase, mice were allowed to explore the apparatus freely for 10 min, with the sliding door open. There were no group preference for either chamber, and the conditioning groups were balanced in an unbiased fashion. The acquisition phase consisted a total of six acquisition trials which were performed in 3 consecutive days, with 2 conditioning trials [Morning trials (8:30–10:30) and afternoon trials (15:30–17:30)] each day. For morning trials, mice were confined to one chamber for 15 min with the presence of a CD1 resident mouse; for afternoon trails, mice were confined to the other chamber for 15 min with the absence of a CD1 resident mouse. On the test day, experimental mice were allowed to explore the apparatus freely for 10 min, with the sliding door open. Behavioral analysis of CPA was performed by calculating the subtracted CPP score (test phase duration in the paired chamber subtracted from the pre-test phase duration in the paired chamber).

### Open Field Test

An acrylic plastic box (40 × 40 × 40 cm^3^) with smooth interior walls was used for the OFT. The center area of the open field (20 × 20 cm^2^) was defined as a centered zone in the arena. Mice were placed in the box and move freely for 10 min. Behaviors was recorded with Viewer animal behavior video tracking software (Biobserve, German). The time duration, number of visits in the centered zone, as well as the total traveled distance were analyzed.

### Elevated Plus Maze Test

The EPM consisted of two open arms (35 × 6 cm^2^) and two enclosed arms (35 × 6 × 15 cm^3^). The open and enclosed walls were connected by a 6 × 6 cm^2^ area. The entire apparatus was elevated 45 cm above the floor level. Mice were brought into the dimly illuminated laboratory 1 h before tests for environmental acclimatization. At the beginning of each trial, mice were placed on the central platform area facing the open arms, and were allowed to explore freely for 5 min. The maze was thoroughly cleaned using a cloth wetted with 75% alcohol after each trial. The percentages of time spent in open arms and enclosed arms were calculated and used to characterize the anxiety level of mice.

### Novel Object Place Recognition Test

The NOPR test consisted of a habituation phase followed by training and testing performed the following day as described in a previously study (Martorell et al., 2019). Twenty-four hours before training, mice were habituated to an open testing arena (40 × 40 × 40 cm^3^) for 5 min. During the training phase, mice were placed into the same box with two identical objects (object−1 and −2) placed in opposite corners. Mice were allowed a total of 20 s of object interaction time (within a maximum time frame of 10 min), and then immediately removed from the arena. Object memory was tested 1 h later using the same procedure as training, except one object was displaced to a novel location. Object exploration was recorded when the snout contacted either object and was calculated by a recognition index (RI), RI = (T_novel_ − T_familiar)_)/(T_novel_ + T_familiar_), where T_novel_ and T_familiar_ indicate the time spent with the novel and familiar location objects, respectively.

### Social Interaction Test

The social interaction test in the present study is to evaluate social avoidance of mice. The test consisted of two sessions. In the first session, the test mouse was allowed to explore an open field arena freely (40 cm × 40 cm) for 5 min. One side of the arena is a wire mesh cage (diameter = 15 cm) that remains empty during the first trial (no target). In the second session, a novel CD1 male mouse was placed into the wire mesh cage, the total time the test mouse spent in the “interaction zone” (an area within 5 cm from the cage) was measured during the 5-min trial (target). The social interaction ratio (SIR) was calculated as follows: SIR = T_target_/T_no__–__target_.

### Prepulse Inhibition Test

PPI tests were conducted in a sound-attenuated box (SR-LAB, Startle Response System, San Diego Instruments, CA, United States). Two sessions were performed in this test: a pulse-alone trial (a 20 ms, 120 dB startle pulse), and a prepulse + pulse trial [a 20 ms prepulse with varying amplitude (75, 80, 85 dB) followed by a 100 ms pause and then the startle pulse]. Briefly, mice were placed in a non-restrictive Plexiglas cylinder mounted on a plastic platform, and their motion was transduced into analog signals via a piezoelectric accelerometer. Before the test, mice were allowed to habituate to a 70-dB background white noise for 5 min, and exposure to the auditory-evoked startle stimuli (120 dB, 20 ms for 10 times). In the PPI test, mice experienced 12 startle trials (120 dB, 20 ms) and 12 prepulse/startle trials (20 ms, white noise at 75, 80, or 85 dB with 100 ms intervals; and 120-dB startle stimulus for 20 ms). Different trial types were presented pseudorandomly, with each trial type presented 12 times. Mouse movement was measured 100 ms after the startle stimulus onset. PPI (%) was calculated as follows: [(startle amplitude on pulse alone trials- startle amplitude on prepulse-pulse trials)/startle amplitude on pulse alone trials] × 100%.

### Statistical Analysis

All data are presented as mean ± SEM. Statistical analyses were performed using GraphPad prism 8.0 (CA, United States). Two-way analysis of variance (ANOVA) was used for NOPR, social avoidance, and startle activity in PPI. The Student’s *t*-test was used to analyze the basic preference and relative preference scores in CPA, locomotion activity in OFT, anxiety level in OFT and EPM, RI in NOPR, and SIR in social avoidance test. Bonferroni-corrected pair-wise comparisons were used as *post hoc* tests. Differences of *p* < 0.05 were considered significant.

## Results

### Experiment Timeline

**FIGURE 1:**
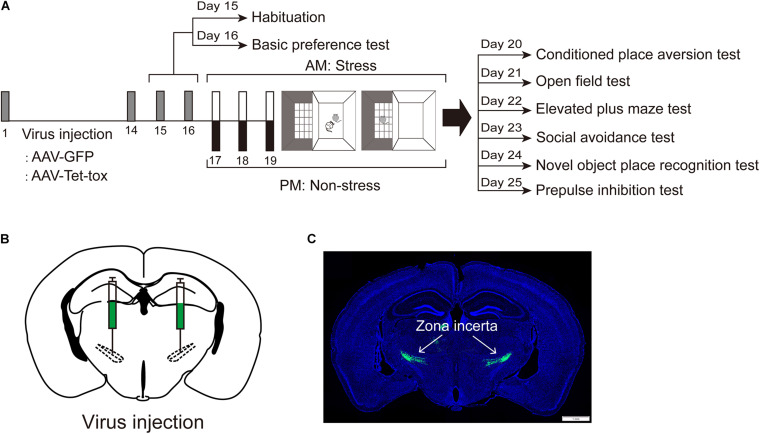
Experiment timeline. **(A)** Experiment timeline. Mice were infected with AAV-GFP or AAV-Tet-tox in the ZI, 2 weeks after the expression of AAV, mice were received a 3 days of conditioned social defeat training. The CPA test, OFT, EPM test, social avoidance test, NOPR test, and PPI test were performed, respectively, after the last training day. **(B,C)** Virus injection sites and expression (Green).

Figure 1A shows the experiment timeline. Mice were randomly divided into 4 groups and were injected with AAV- GFP or AAV-Tet-tox, respectively, according to the grouping described in materials and methods section. Two weeks after recovery from surgery, mice were allowed to freely explore the CPA apparatus for 10 min of habituation, followed by a 10 min of basic preference test in the next day. Then mice were experienced 3 consecutive days of RSDS training for the acquisition of social CPA. The expression of CPA, as well as the locomotion activity, anxiety level, spatial cognition, social avoidance, and PPI were evaluated. Figures 1B,C show the virus injection sites and expression.

### Zona Incerta Silencing Inhibits Repeated Social Defeat Stress-Induced Social Conditioned Place Aversion

**FIGURE 2:**
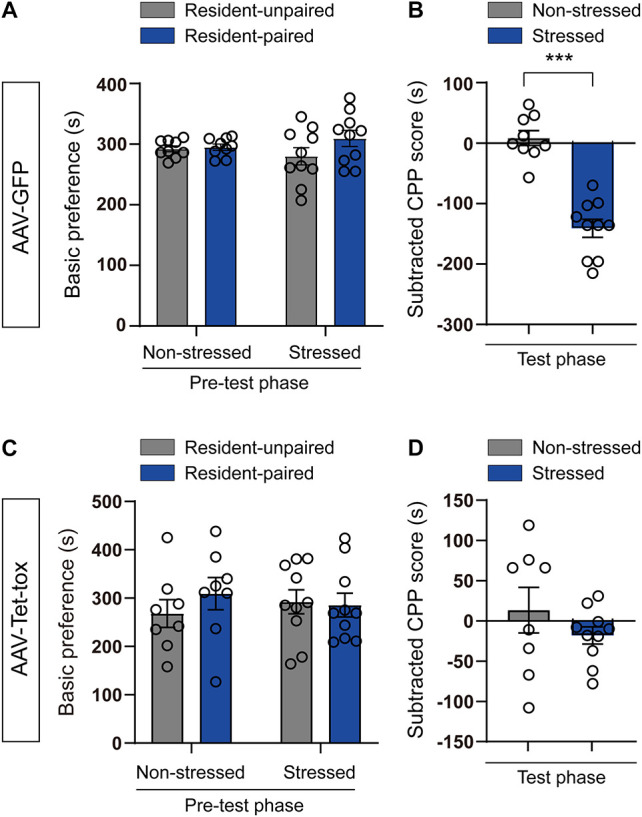
Effect of ZI silencing on RSDS-induced CPA. **(A,C)** The basic preference of mice for the CPA apparatus were examined in pre-test phase. The mice in all groups did not exhibit an innate preference for the CPA apparatus. The two-tailed paired *t*-test was used to compare the preference score of mice in resident-unpaired and resident paired chambers. **(B,D)** The subtracted CPP score was recorded in test-phase. The stressed mice in AAV-GFP group showed marked aversive effects to the resident-paired chamber, whereas the stressed mice in AAV-Tet-tox group showed less marked aversive effects. The two-tailed unpaired *t*-test was used to compare the subtracted CPP score of mice in non-stressed and stressed groups. ***P < 0.001.

To investigate the role of ZI in social CPA, an aggression-based CPA procedure was developed via combination of the resident-intruder and CPA paradigms. In this model, C57BL/6J intruder mice received conditioned aversive stimulation from CD1 mice in CPA apparatus to acquire CPA. Before CPA training, mice did not exhibit an innate preference for the CPA apparatus (Figures 2A,C). After 3 days of CPA training, the subtracted CPP score of the stressed mice were significantly decreased compared to the non-stressed mice in control AAV-GPF group (*t* = 7.602, df = 17, *p* < 0.001; Figure 2B). ZI silencing completely blocked the expression of CPA in AAV-Tet-tox group (*t* = 1.128, df = 16, *p* = 0.275; Figure 2D). This result indicated that the activity of ZI neuron may involve in the formation of RSDS-induced social CPA.

### Zona Incerta Silencing Induces Anxiety and Spatial Cognitive Impairment in Repeated Social Defeat Stress-Treated Mice

**FIGURE 3:**
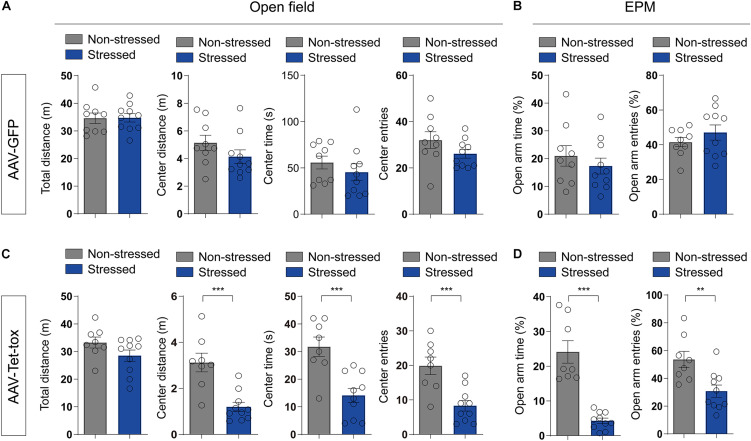
Effect of ZI silencing on locomotion activity and anxiety. **(A,C)** The total distance in open filed, and the distance, time and number of entries in the middle area of the open field were recorded in mice. The stressed mice in AAV-GFP group did not show marked changes in locomotion activity and anxiety level, whereas the stressed mice in AAV-Tet-tox group showed marked anxiety-like behavior, but had no change in locomotion activity. The two-tailed unpaired *t*-test was used to compare the behavioral parameters of OFT in non-stressed and stressed groups. **(B,D)** The percentages of time and entries in the open arms of EPM were recorded in mice. The stressed mice in AAV-GFP group did not show marked changes in anxiety level, whereas the stressed mice in AAV-Tet-tox group showed marked anxiety-like behavior. The two-tailed unpaired *t*-test was used to compare the behavioral parameters of EPM test in non-stressed and stressed groups. **P < 0.01, ***P < 0.001.

**FIGURE 4:**
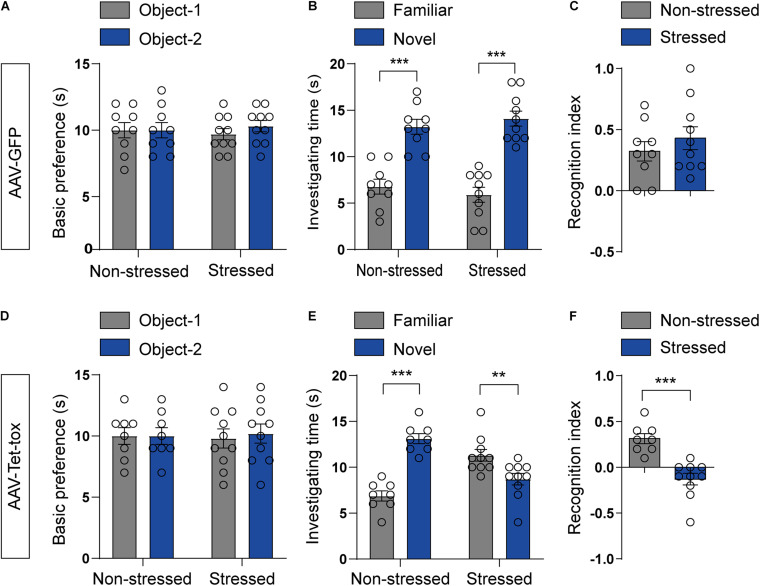
Effect of ZI silencing on spatial cognitive ability. **(A,D)** The basic preference of mice for two identical objects were examined before training. The mice in all groups did not exhibit an innate preference for the two identical objects. The two-tailed paired *t*-test was used to compare the preference score of mice for object-1 and object-2. **(B,E)** The investigating time of mice for two identical objects were examined after training. The non-stressed mice spent more investigating time on the novel location object than that on the familiar location object, whereas the stressed mice spent more investigating time on the novel location object only in AAV-GFP group, and showed the opposite phenotype in AAV-Tet-tox group. Two-way ANOVA with bonferroni *post hoc* test was used to compare the investigating time for the novel and familiar location object. **(C,F)** The RI of two identical objects by mice was recorded after training. The stressed mice showed similar RI to the non-stressed mice in AAV-GFP group, while the RI was significantly decreased in stressed mice compared to the non-stressed mice in AAV-Tet-tox group. The two-tailed unpaired *t*-test was used to compare the RI between non-stressed and stressed mice. **p < 0.01, ***p < 0.001.

RSDS usually induces PTSD-like behaviors, such as anxiety, cognition disorder, social avoidance, and PPI deficit (Huang et al., 2016; Yang et al., 2018; Ishikawa et al., 2019). We examined the locomotion activity and anxiety level using open filed and EPM test, respectively. As illustrated in Figure 3, RSDS under the current procedure has no effect on the locomotion activity and anxiety level. RSDS did not affect the total movement distance in open filed (*t* = 0.053, df = 17, *p* = 0.958), the movement distance (*t* = 1.371, df = 17, *p* = 0.188), time spent (*t* = 0.896, df = 17, *p* = 0.382), and number of entries (*t* = 1.502, df = 17, *p* = 0.151) of mice in the middle area of open field (Figure 3A). RSDS also did not affect the percentage of time spent in the open arm (*t* = 0.795, df = 17, *p* = 0.437) and the percentage of number of entries into the open arm (*t* = 1.044, df = 17, *p* = 0.311) of EPM (Figure 3B). However, ZI silencing significantly decreased the movement distance (*t* = 4.526, df = 16, *p* < 0.001), time spent (*t* = 4.176, df = 16, *p* < 0.001), and number of entries (*t* = 4.071, df = 16, *p* < 0.001) of RSDS-treated mice in the middle area of the open field (Figure 3C), and also decreased the percentage of the time the mice spent in the open arm (*t* = 6.610, df = 16, *p* < 0.001) and the percentage of the number of entries into the open arm (*t* = 3.119, df = 16, *p* = 0.005)of EPM (Figure 3D), but had no effect on the total movement distance in open field compared to the non-stressed mice (*t* = 1.634, df = 16, *p* = 0.121) (Figure 3C). This result suggested that ZI silencing promoted anxiety but had no effect on locomotion activity in stressed mice. We also evaluated the spatial cognition with NOPR test in mice, the result showed that the basic preference for the two identical objects in different group mice had no significant difference before training (Figures 4A,D). The non-stressed mice spent more time with the novel location object in NOPR test, whereas the stressed mice that infected with AAV-Tet-tox in the ZI, showed the opposite behavior compared to the mice that infected with AAV-GFP (Figures 4B,E). Two-way ANOVA revealed a significant location × stress interaction [*F*_(1, 32)_ = 52.57, *p* < 0.001], bonferroni *post hoc* test showed significant difference between the investigating time for familiar and novel location in non-stressed (*p* < 0.001, Figures 4B,E) and stressed mice (*p* < 0.001, Figure 4B; *p* = 0.006, Figure 4E). RSDS did not affect the RI of GFP mice to the novel and familiar location objects (*t* = 0.869, df = 17, *p* = 0.396, Figure 4C), while ZI silencing significantly decreased the recognition ability to different location object (*t* = 5.127, df = 16, *p* < 0.001, Figure 4F). This result indicated that ZI-silencing impaired the spatial cognitive ability of stressed mice, but had no effect on the non-stressed mice.

### Zona Incerta Silencing Inhibits Social Avoidance and Prepulse Inhibition Impairment Induced by Repeated Social Defeat Stress

**FIGURE 5:**
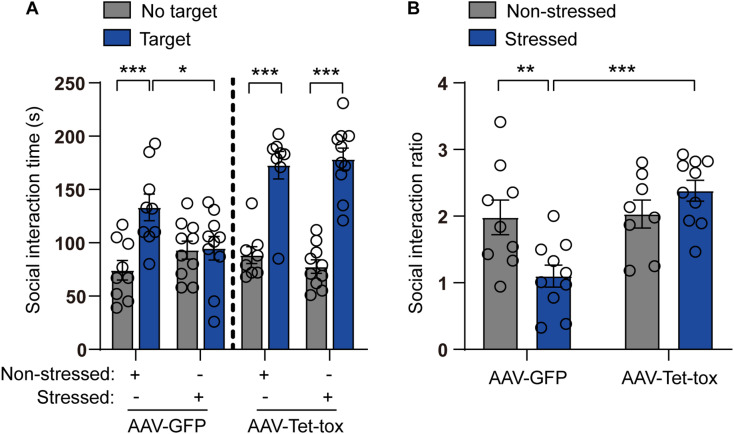
Effect of ZI silencing on RSDS-induced social avoidance. **(A)** Social interaction time of mice was examined after RSDS training. In the AAV-GFP group, the non-stressed mice showed significant social interaction when the target mice were present, while the stressed mice showed less social interaction; in the AAV-Tet-tox group, both the non-stressed and stressed mice showed significant social interaction when the target mice were present. Two-way ANOVA with bonferroni *post hoc* test was used to compare the social interaction time between target and no target group. **(B)** SIR of mice was examined after RSDS training. The SIR of stressed mice was significantly decreased compared to the non-stressed mice in AAV-GFP group, while it was reversed in AAV-Tet-tox group. Two-way ANOVA with bonferroni *post hoc* test was used to compare the SIR of the non-stressed and stressed mice in AAV-GFP group, and was also used to compare SIR of the stressed mice in AAV-GFP and AAV-Tet-tox group. *p < 0.05, **p < 0.01, ***p < 0.001.

**FIGURE 6:**
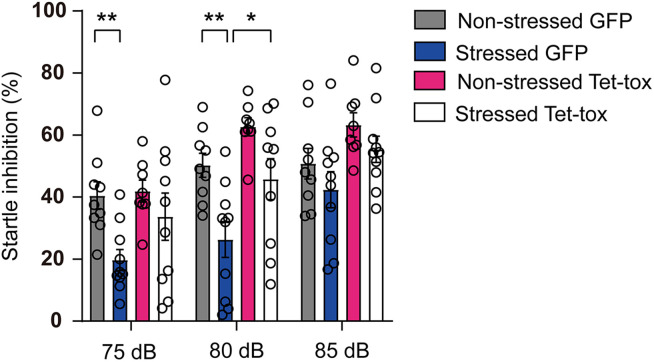
Effect of ZI silencing on RSDS-induced PPI deficit. The startle inhibition ratio of mice was examined after RSDS training. The stressed GFP mice showed a significantly lower startle inhibition rate than the non-stressed GFP mice in 75 and 80 dB groups, while the startle inhibition rate was significantly increased in the stressed Tet-tox mice compared to the stressed GFP mice in 80 dB group. Two-way ANOVA with bonferroni *post hoc* test was used to compare the startle inhibition rate of non-stressed and stressed GFP mice, and was also used to compare the startle inhibition rate of stressed GPF mice and stressed Tet-tox mice. *p < 0.05, **p < 0.01.

As shown in Figure 5, the social avoidance propensity of mice was evaluated with the test of social interaction task, and the result showed that the social interaction time and ratio of non-stressed mice in the presence of target mice were significantly increased compared with that in the absence of the target mice, and this social activity was markedly decreased in stressed mice in AAV-GFP group but not AAV-Tet-tox group, when compared with the non-stressed mice. The result indicated that the stressed mice exhibited social avoidance, which can be reversed by ZI silencing. Two-way ANOVA revealed a target × stress interaction [AAV-GFP group: *F*_(1, 34)_ = 7.593, *p* = 0.009; AAV-Tet-tox group, *F*_(1, 32)_ = 0.735, *p* = 0.397], bonferroni *post hoc* test showed significant difference between no target and target in non-stressed GFP group (*p* < 0.001), and in non-stressed (*p* < 0.001) and stressed (*p* < 0.001) Tet-tox groups; bonferroni *post hoc* test also showed significant difference between non-stressed and stressed mice in AAV-GFP group (*p* = 0.268) (Figure 5A). For Figure 5B, two-way ANOVA revealed a significant AAV treatment × stress interaction [*F*_(1, 33)_ = 9.511, *p* = 0.004], bonferroni *post hoc* test showed significant difference between the non-stressed and stressed mice in AAV-GFP group (*p* = 0.006); bonferroni *post hoc* test also showed significant difference between the AAV-GFP group and AAV-Tet-tox group in stressed mice (*p* < 0.001) (Figure 5B). As shown in Figure 6, RSDS induced marked impairment in PPI in AAV-GFP group mice, while ZI-silencing blocked the RSDS induced impairment in stressed mice. Two-way ANOVA revealed AAV treatment × stress interactions [75 dB: *F*_(1, 33)_ = 1.420, *p* = 0.242; 80 dB: *F*_(1, 33)_ = 0.439, *p* = 0.512; 85 dB: *F*_(1, 33)_ = 0.002, *p* = 0.959], bonferroni *post hoc* test showed significant difference between the non-stressed and stressed GPF mice in 75 dB (*p* = 0.023) and 80 dB (*p* = 0.006) groups; bonferroni *post hoc* test also showed significant difference between the stressed GPF mice and stressed Tet-tox mice in 80 dB group (*p* = 0.031).

## Discussion

In this study, we established a CPA paradigm by using aggression training procedure based on the theory of Pavlovian learning, to investigate the effect of ZI on social CPA, as well as on PTSD-like behaviors. We showed that 3 episodes of SD significantly induced the expression of social CPA which was blocked by ZI silencing. Meanwhile, RSDS induced several phenotypes of PTSD including social avoidance and PPI deficit in mice in control AAV-GFP group, but did not cause anxiety and spatial cognitive disorder. ZI silencing decreased social avoidance and blocked PPI deficit, and also promoted anxiety and spatial cognitive impairment in stressed mice.

Multiple aversive stimulus, such as foot shock, plantar incision, predator odor, chemical reagents, chemotherapeutic agents, addictive drug, and social defeat, have been reported to induce aversion-associated behaviors including CPA, defense, conditioned fear, and fear generalization (Takahashi et al., 2008; Sugiyama et al., 2017; Curry et al., 2018; Bian et al., 2019; Cooper et al., 2021). ZI has been widely implicated in the aversion-associated behaviors. Previous study has shown that inactivation of the medial ZI strongly impaired fear conditioning (tone-foot shock) via blocking the initial acquisition of fear memory and the remote retrieval of previously acquired fear memory; silencing of ZI parvalbumin-positive (PV^+^) neurons but not the tyrosine hydroxylase-positive (TH^+^) neurons and somatostatin-positive (SST^+^) neurons showed similar results (Zhou et al., 2018), indicating that ZI was involved in the modulation of fear conditioning memory, and the ZI PV^+^ neurons are uniquely essential for the acquisition of fear conditioning memory and the remote retrieval. Our result showed that ZI silencing blocked the acquisition of social fear conditioning memory, indicating that the ZI modulation of fear conditioning memory was independent of the stimuli type. However, whether silencing of ZI PV^+^ neurons can reproduce the behavioral disorder caused by silencing all ZI neurons needs to be further explored. The TH^+^ neurons of ZI are suggested to modulate the responses to aversive stimuli (Essig and Felsen, 2016), while the TH^+^ neurons of the ventral tegmental area (VTA) are sensitive to aversive contexts. Besides, repeated exposure to aversive contexts (context-social aggression) is necessary to activate the VTA TH^+^ neurons in females (Greenberg et al., 2015). The TH^+^ neurons, therefore, may involve in the modulation of social fear conditioning memory, although such a role has not been observed in tone-foot shock fear conditioning (Zhou et al., 2018).

In addition to the social CPA, we also examined the PTSD-like behaviors that have been widely reported to be induced by RSDS, including anxiety, spatial cognitive disorder, social avoidance, and PPI deficit (Desbonnet et al., 2012; Pfau and Russo, 2016; McCullough et al., 2021; Torres-Berrío et al., 2021), to evaluate the effect of ZI on PTSD-like behaviors and further explain the behavioral mechanism underlying ZI modulation of social fear conditioning memory. We first examined the locomotion activity which may influent the expression of CPA, the result showed that both RSDS and ZI silencing did not affect the locomotion activity. This indicated that the impaired social fear conditioning memory was not associated with locomotion activity. ZI silencing, however, increased anxiety and resulted in spatial cognitive impairment in stressed mice, although the RSDS alone had not effect on anxiety and spatial cognitive ability in control AAV-GFP group mice. This indicated that the 3 episodes of SD were not enough to induce anxiety disorder and spatial cognitive impairment, and ZI silencing may enhance the stress sensitivity of anxiety and spatial cognition in mice. Previous studies have shown that anxiety increases the place conditioning induced by cocaine in rats (Pelloux et al., 2009), and the prevention of CPA is accompanied by the impairment of spatial cognition (Shi et al., 2019). This indicated that anxiety increase and spatial cognitive impairment possess the potential to influence the expression of place conditioning behavior, and the increased anxiety and impaired spatial cognitive ability may be responsible for the impaired CPA behavior. Furthermore, we found that ZI silencing blocked RSDS-induced social avoidance and PPI deficit in stressed mice. Previous studies on the ZI modulation of PTSD-like behaviors mainly focused on fear conditioning memory, fear generalization and extinction learning deficit. Activation of ZI or targeted stimulation of GABAergic cells in the ZI attenuates fear generalization and enhances extinction recall. Targeted activation of the dopaminergic ZI projection to thalamic reunions has no effect on fear generalization but enhances extinction recall in a dopamine receptor D1-dependent manner (Venkataraman et al., 2019, 2021). Silencing of ZI or specifically suppression of the ZI PV^+^ neurons blocks fear conditioning memory acquisition and remote retrieval (Zhou et al., 2018). There was no report, however, about the role of ZI in modulating RSDS-induced social avoidance and PPI deficit. Therefore, we suggested that ZI may possess the potential to modulate social fear conditioning memory and PPI in addition to modulating tone-foot shock fear conditioning memory.

## Conclusion

A mouse model of social CPA was established in the present study. In RSDS-treated mice, ZI silencing displayed an inhibitory effect on social CPA, social avoidance, and PPI deficit; and a facilitative effect on anxiety and spatial cognitive impairment. We proposed that the activity changes of the GABAergic and dopaminergic neurons in the ZI may be responsible for the deficit of social CPA and the expression of PTSD-like behaviors. The precise neurobiological mechanism, however, still needed to be further explored. More future works, therefore, need to be focused on the identification of GABAergic and dopaminergic neuron types and their projection circuits in the ZI that involve in the modulation of social CPA and PTSD-like behaviors.

## Data Availability Statement

The original contributions presented in the study are included in the article/supplementary material, further inquiries can be directed to the corresponding author/s.

## Ethics Statement

The animal study was reviewed and approved by the Institutional Animal Care and Use Committee of the Huazhong University of Science and Technology.

## Author Contributions

MH and HZ designed the research and analyzed the data. MH, HZ, and WX performed the experiments, including virus injection, behavioral test, and histological analysis. MH drafted the manuscript. MH and WX provided a critical revision of the manuscript. All authors contributed to and approved the final version.

## Conflict of Interest

The authors declare that the research was conducted in the absence of any commercial or financial relationships that could be construed as a potential conflict of interest.

## Publisher’s Note

All claims expressed in this article are solely those of the authors and do not necessarily represent those of their affiliated organizations, or those of the publisher, the editors and the reviewers. Any product that may be evaluated in this article, or claim that may be made by its manufacturer, is not guaranteed or endorsed by the publisher.
